# The Incidental Discovery of an Ileal Conduit Calculus: A Case Report

**DOI:** 10.7759/cureus.43299

**Published:** 2023-08-10

**Authors:** Colby Kihara, Arsh N Patel, Katie Oakley, Carter Gay, Laurence Stolzenberg, Jason Seale

**Affiliations:** 1 Research, Alabama College of Osteopathic Medicine, Dothan, USA; 2 Medicine, Alabama College of Osteopathic Medicine, Dothan, USA; 3 Orthopedic Surgery, Alabama College of Osteopathic Medicine, Dothan, USA; 4 General Surgery, Decatur Morgan Hospital, Decatur, USA

**Keywords:** small bowel obstruction, small bowel necrosis, total cystectomy, gallstone ileum, bladder calculus, reconstructive urology and medical education, emergent general surgery

## Abstract

In patients who have undergone radical cystectomy, urinary diversion procedures such as ileal conduits may pose a considerable problem for urologists when they result in stone formation. While an ileal conduit stone is only one of many potential complications of this procedure, its management may be particularly challenging; symptoms and treatments vary depending on factors such as stone location, composition, and the patient's BMI. We present a unique case of a 91-year-old male with a history of prostate and bladder cancer status post-radical prostatectomy, cystectomy, and ileal conduit who presented to the emergency department complaining of abdominal pain, nausea, vomiting, and disorientation for the last 24 hours. The patient was diagnosed with a complete bowel obstruction secondary to gallstone ileus. Consequently, an exploratory laparotomy was performed, which revealed that his small bowel obstruction was not caused by gallstone ileus but rather secondary to an internal hernia and volvulus associated with a previous bowel resection and anastomosis. The stone that was originally thought to be causing the small bowel obstruction turned out to be a 3.3 cm stone in his ileal conduit, which ultimately had no part in causing the patient's small bowel obstruction. Calculus formation is an infrequent complication of ileal conduit placement. Our aim in presenting this case is to increase awareness of this rare complication that can arise without symptoms with the hopes of increasing early intervention and improving outcomes.

## Introduction

An ileal conduit is a form of urinary diversion that is often used in the management of patients who have undergone a radical cystectomy. Since its development in the 1950s, the procedure has become a standard of treatment, especially in elderly patients and individuals with anatomical constraints, limited dexterity, poor motivation, and poor renal function [1]. Complications are wide-ranging but commonly include parastomal herniation, bowel dysfunction, nutrient malabsorption, vitamin B12 deficiency, renal insufficiency, urinary tract infections, and hyperchloremic metabolic acidosis [2,3]. Calculus formation is a less common complication that develops in approximately 3.5% of patients with an ileal conduit [4]. The underlying mechanism is thought to be caused by several factors such as the solubility of compounds in alkaline urine, dietary malabsorption, the presence of intestinal mucus in the conduit, and possibly retained sutures or staples serving as a nidus for stone formation [2]. We present a case of large ileal conduit calculi and its successful management through an open surgical procedure.

## Case presentation

**Figure 1:**
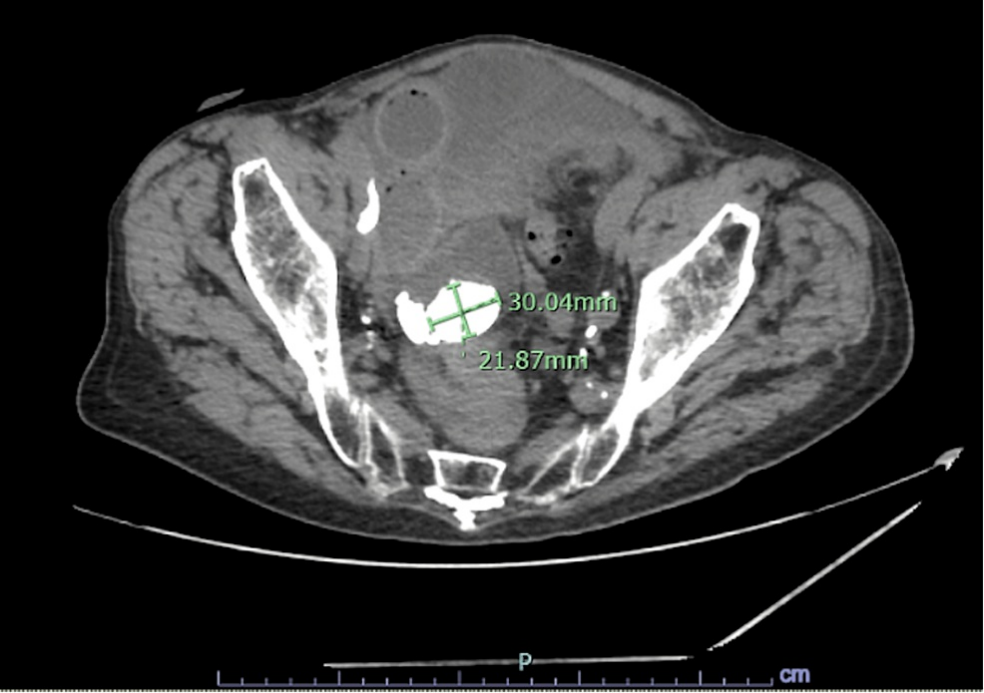
Abdominal and pelvic CT scan showing a 3 cm stone located in a distended loop of the small bowel in the pelvis CT: computed tomography

**Figure 2:**
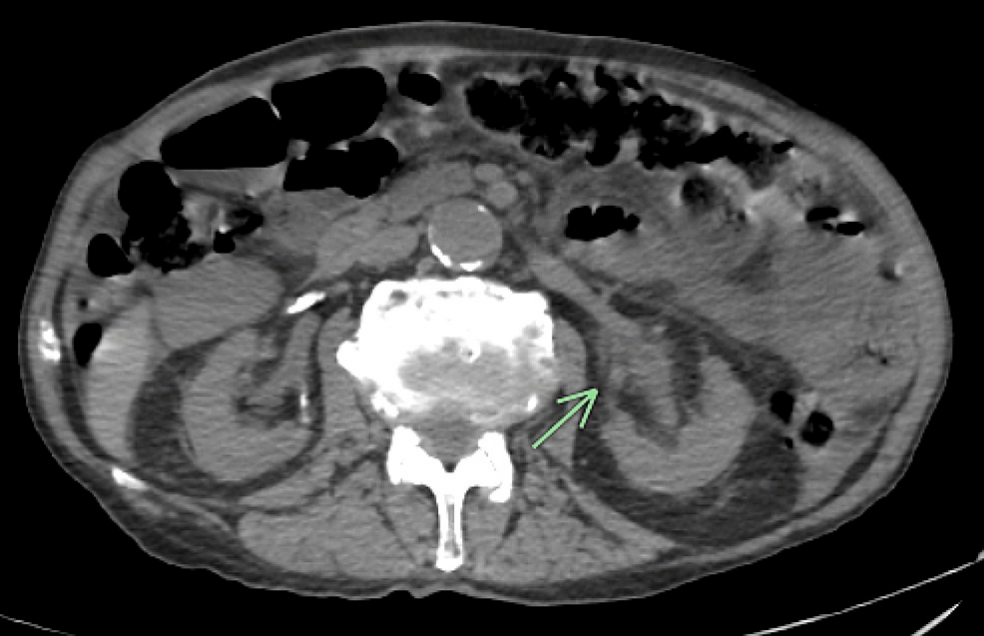
Abdominal and pelvic CT scan showing left hydronephrosis and hydroureter with the thickening of the renal pelvic mucosa CT: computed tomography

**Figure 3:**
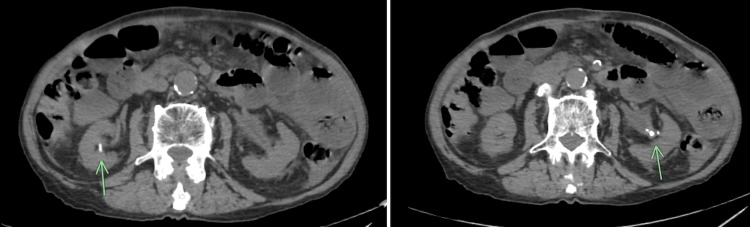
Abdominal and pelvic CT scan showing right and left nephrolithiasis CT: computed tomography

**Figure 4:**
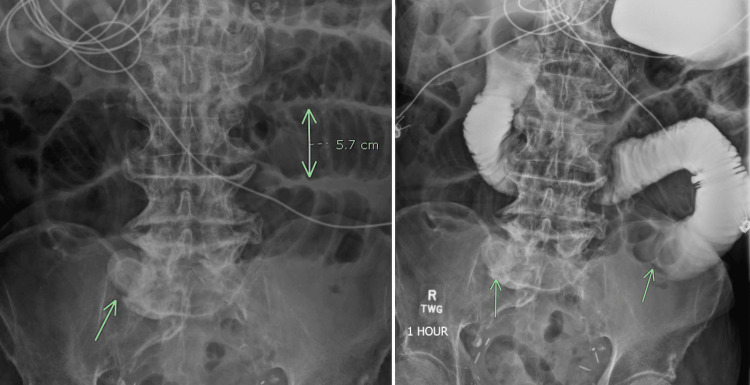
Small bowel series The image on the left demonstrates a stone with proximal small bowel consistently dilated up to 5.7 cm (normal is 3 cm). The right image demonstrates a stone and the area of obstruction proximal to the stone

A 91-year-old male with a history of prostate and bladder cancer status post-radical prostatectomy, cystectomy, and ileal conduit presented to the emergency department with a chief complaint of abdominal pain, nausea, vomiting, and disorientation for the last 24 hours. The patient stated that the abdominal pain had begun roughly two weeks prior and had gotten progressively worse. The patient's family was at the bedside in the emergency department and reported observing coffee-ground emesis. The patient also reported a few episodes of melena in the preceding two days. On physical examination, the bedside monitor reported the following vital signs: temperature of 98°F, heart rate of 50 beats per minute, respiratory rate of 14 breaths per minute with 98% oxygen saturation, and blood pressure of 132/88 mmHg. As the abdomen was examined, the patient demonstrated mild diffuse tenderness to palpation with no radiation of pain. All other systems were negative. The patient's heart rate appeared to be irregular, so a 12-lead electrocardiogram (ECG) was performed. The ECG revealed possible atrial fibrillation, which was resolved with the administration of fluids in the emergency department. A computed tomography (CT) scan of the abdomen and pelvis revealed a large stone located in a distended loop of the small bowel of the pelvis (Figure 1), left hydronephrosis and hydroureter with the thickening of the renal pelvic mucosa (Figure 2), bilateral nephrolithiasis (Figure 3), ascites, and subsegmental atelectasis. Initial laboratory results revealed a urinary tract infection (Table 1) with an acute kidney injury (Table 2). The patient was admitted to the ward and placed on nothing by mouth (NPO) restrictions. The patient was then taken to complete a small bowel series, which revealed a high-grade distal small bowel obstruction and a severe gallstone ileus likely causing the bowel obstruction. This was determined based on the location of the stone and the dilated small bowel that appeared to be proximal to the stone (Figure 4). General surgery was consulted, and on hospital admission day 2, the patient was taken for an exploratory laparotomy.

**Table 1:** Urine analysis on admission HPF, high-power field; TNTC, too numerous to count

Urine analysis	Collected on admission
Urine color	Orange
Urine turbidity (reference: clear)	Turbid
Urine pH	8.5
Urine specific gravity	1.011
Urine protein (reference: negative)	200 mg/dL
Urine glucose (reference: negative)	Negative
Urine ketones (reference: negative)	10 mg/dL
Urine blood (reference: negative)	Moderate
Urine leukocytes (reference: negative)	Large
Urine WBC (reference: <10/HPF)	TNTC
Urine RBC (reference: <10/HPF)	10-20
Urine nitrite (reference: negative)	Negative
Urine bacteria	3+
Urine bilirubin (reference: negative)	Negative
Urine casts	None seen
Urine epithelial cells (reference: <10/HPF)	Negative
Urine yeast	None seen

**Table 2:** Complete blood count and basic metabolic panel results on admission Hgb, hemoglobin; Hct, hematocrit; BUN, blood urea nitrogen; GFR, glomerular filtration rate; AST, aspartate aminotransferase; ALT, alanine transaminase

	Collected on admission	Collected on day of discharge
Hgb	13.7 g/dL (reference range: 14.0-18.0 g/dL)	10.3 g/dL
Hct	39.2% (reference range: 42.0%-52.0%)	31.0%
RBC	4.33 XMIL (reference range: 4.7-6.1 XMIL)	3.30 XMIL
WBC	18.33 × 1000 (reference range: 4.8-10.8 × 1000)	11.50 × 1000
Sodium	141 mmol/L (reference range: 136-145 mmol/L)	144 mmol/L
Potassium	4.7 mmol/L (reference range: 3.5-5.1 mmol/L)	3.9 mmol/L
Chloride	101 mmol/L (reference range: 98-107 mmol/L)	114 mmol/L
Calcium	9.9 mg/dL (reference range: 8.8-10.2 mg/dL)	8.8 mg/dL
Phosphorus	1.5 mg/dL (reference range: 2.7-4.5 mg/dL)	1.8 mg/dL
Carbon dioxide	24 mmol/L (reference range: 25-35 mmol/L)	21 mmol/L
Anion gap	16	9
BUN	38 mg/dL (reference range: 8-22 mg/dL)	39 mg/dL
Creatinine	1.4 mg/dL (reference range: 0.7-1.2 mg/dL)	1.1 mg/dL
Estimated GFR	48 mL/minute/1.73 m^2^	>60 mL/minute/1.73 m^2^
Total bilirubin	1.41 mg/dL (reference range: 0.20-1.00 mg/dL)	0.85 mg/dL
Direct bilirubin	0.30 mg/dL (reference range: 0.00-0.20 mg/dL)	0.1 mg/dL
AST	14 U/L (reference range: 10-34 U/L)	15 U/L
ALT	9 U/L (reference range: 10-44 U/L)	8 U/L
Alkaline phosphatase	90 U/L (reference range: 32-122 U/L)	61 U/L
Total protein	7.6 g/dL (reference range: 6.3-8.3 g/dL)	5.9 g/dL
Albumin	4.4 g/dL (reference range: 3.5-5.0 g/dL)	2.6 g/dL

During the intraoperative exploration, the patient was found to have a closed-loop small bowel obstruction secondary to an internal hernia and small bowel volvulus. These findings were notably associated with the patient's previous history of bowel resection and anastomosis associated with the placement of the ileal conduit. This caused 36 cm of small bowel necrosis. The necrotic bowel was removed, and the two ends of the healthy small bowel were anastomosed. Upon further inspection, the patient did not have gallstone ileus. What was originally thought to be a gallstone ileus actually turned out to be a 3.3 cm stone in the patient's ileal conduit. The stone was freely mobile and easily removed through enterotomy. Following the completion of the surgery, the patient was closely monitored for any post-operative complications. Gradual oral intake was initiated, starting with clear liquids on post-operation day 2, which was then advanced to a full clear liquid diet on post-operation day 3. Notably, on post-operation day 4, the patient exhibited the passage of flatus, prompting the advancement of the diet to a soft gastrointestinal (GI) diet. Concurrently, the patient's acute kidney injury demonstrated resolution with appropriate fluid administration. The patient reported a bowel movement post-operation day 6 and was discharged in stable condition with the appropriate medications, return of normal bowel function, and no abdominal pain or discomfort.

## Discussion

An ileal conduit procedure involves the resection of an ileal segment followed by anastomosis to the ureters and exteriorization to allow urine drainage and excretion. While historically considered to be the gold standard method of urinary diversion for patients who have undergone radical cystectomy, ileal conduits present with a wide range of potential complications. The most common is parastomal or incisional herniation, which can occur in up to 65% of patients and requires surgical intervention in as many as 30% of cases [5]. Furthermore, many other associated complications can result from ileal resection and subsequent anastomosis such as bowel dysfunction and nutrient deficiencies. Electrolyte abnormalities are also common and can predispose the patient to metabolic acidosis and arrhythmias. Additionally, the exteriorization of the stoma increases the frequency of urinary tract infections, as well as the development of renal insufficiency. Lastly, while rare, the presence of a fistula between the small bowel and the ileal conduit could allow the passage of intestinal contents into the conduit and vice versa [6].

A less well-known complication associated with ileal conduit urinary diversion is the formation of large calculi, which only occur in 3.5% of patients [6]. While most urolithiases reported in the literature appear to present in the upper urinary tracts, there have been several cases of ileal conduit stones reported in the literature [7]. Alkaline urine, the presence of intestinal mucus, and dietary malabsorption are common in patients with ileal conduits and serve as risk factors for the formation of calculi in the ileal conduit [3]. In the literature, it is reported that most ileal conduit stones are primarily composed of magnesium ammonium phosphate (struvite) [8], which was the case in our patient. However, other stones have been reported as being composed of calcium oxalate, calcium phosphate, hydrogen urate, and/or carbonate apatite [9]. The presence of calculi in the conduit could lead to urinary outlet obstruction with resultant urinary tract infection and acute kidney injury, both of which were seen in this patient.

The treatment of urinary calculi in patients with ileal conduits is based on the patient's symptoms and the characteristics of the stone (size, location, and composition) [5]. If surgery is deemed necessary, there are several percutaneous-based procedures such as percutaneous nephrolithotomy, antegrade ureteroscopy with semirigid ureteroscope or flexible ureteroscope from the percutaneous tract, and percutaneous pouch lithotripsy, all of which provide safe access to target stones limiting postoperative complications [10].

## Conclusions

Calculus formation within an ileal conduit is an uncommon sequela that may develop in patients who have undergone radical cystectomy. In this case, a large ileal conduit calculus was incidentally discovered in a patient who presented with signs and symptoms of a small bowel obstruction. The small bowel obstruction was visualized on CT imaging and mistaken for a gallstone ileus, which in turn led to an exploratory laparotomy and the discovery of the ileal conduit calculus. This case report serves to highlight the need to include ileal conduit calcification in the differential diagnosis for adynamic ileus due to the risk for complications that can develop if not diagnosed and addressed appropriately.
